# How Valid Are Wearable Devices in Team Sports? A Systematic Review

**DOI:** 10.3390/sports14070264

**Published:** 2026-06-26

**Authors:** Nebojša Čokorilo, Nikola Manolopoulos, Tamara Matijević, Ranko Rajović

**Affiliations:** 1 Faculty of Sport and Physical Education, University of Novi Sad, 21000 Novi Sad, Serbia; nikolamanolopoulos@gmail.com (N.M.); tamaramatijevic99@gmail.com (T.M.); 2 Faculty of Pedagogy, University Primorska, 6000 Koper, Slovenia

**Keywords:** physiological monitoring, team sport athletes, heart rate, energy expenditure, VO_2_max, inertial sensors, validation studies

## Abstract

The aim of this systematic review was to evaluate the validity and accuracy of wearable technologies used for monitoring physiological metrics in team-sport athletes. A systematic literature search was conducted in PubMed and Scopus databases, with additional studies identified through supplementary searching. Studies published between 2015 and 2025 were included if they assessed wearable devices in team-sport populations and compared their measurements with gold-standard methods. A total of eleven studies met the inclusion criteria. The findings indicate that heart rate monitoring demonstrates consistently high validity across different wearable devices, particularly in controlled laboratory conditions. In contrast, energy expenditure estimation shows substantial variability and systematic underestimation, especially during high-intensity and intermittent activities typical of team sports. VO_2_max estimation presents mixed validity depending on device type and testing protocol, while respiratory frequency measurement demonstrates high agreement with gold-standard methods when assessed using specialized devices. Overall, wearable technologies provide valuable insights into athlete monitoring; however, their accuracy varies considerably depending on the physiological parameter and testing environment. These findings highlight the need for improved validation protocols and caution in the application of wearable-derived data in high-performance team-sport settings.

## 1. Introduction

Wearable technology has emerged as one of the most influential innovations in contemporary sports science, reshaping the ways in which performance, physiological responses, and biomechanical demands are monitored across athletic populations. Over the past two decades, the accessibility and sensor complexity of wearable devices have expanded dramatically, allowing practitioners to quantify multiple dimensions of athlete behavior in real time [1,2]. These advancements have facilitated a transition from basic activity tracking to sophisticated multi-sensor systems capable of capturing granular metrics related to movement, internal load, collision events, and environmental context [3,4].

Correspondingly, sports scientists, coaches, and medical teams have increasingly integrated wearable technologies into daily training and competitive environments, aiming to enhance performance optimization, injury prevention strategies, and tactical understanding [5,6]. The widespread adoption of wearable systems is particularly notable in team sports, where the dynamic and intermittent nature of gameplay creates a compelling need for accurate load monitoring. Team sports are characterized by frequent accelerations, decelerations, abrupt changes in direction, high-intensity sprints, and collision-based movements, all performed under conditions of situational pressure and tactical complexity [7,8].

The dynamic and intermittent movement patterns characteristic of team sports present substantial challenges for sensor validity, as locomotor demands often exceed the controlled conditions under which many devices are originally validated. Early findings have demonstrated that global positioning system (GPS) technology, despite its widespread use, exhibits notable limitations in measuring short-distance sprints, rapid velocity fluctuations, and non-linear trajectories. These movement characteristics are fundamental components of match play in sports such as soccer, rugby, basketball, and handball [9,10].

In applied settings, wearable technologies are widely used in team sports such as soccer, basketball, rugby, and volleyball to monitor external load and performance metrics. However, the complex and unpredictable nature of these sports often reduces measurement accuracy, particularly during multidirectional movements, player congestion, and rapid transitions between intensities. Accordingly, several studies have reported that wearable sensors may fail to produce accurate outputs under these ecologically valid conditions [11,12].

Challenges related to measurement validity extend beyond external locomotor tracking. Internal load monitoring via wearable devices, including heart rate and heart rate variability (HRV) sensors, demonstrates variable accuracy when compared with electrocardiography or laboratory-grade equipment, particularly during high-intensity and rapidly changing exercise conditions [13,14]. Similarly, inertial measurement units (IMUs), which hold significant promise due to their ability to capture high-frequency accelerations, rotational velocities, and joint kinematics, remain vulnerable to cumulative drift, magnetic interference, and algorithmic uncertainty [4,15]. These issues are further magnified in the ecologically dynamic settings of team sports, where complex movement patterns and unpredictable interactions between players introduce additional layers of biomechanical noise [16].

Taken collectively, current evidence underscores that although wearable technologies provide valuable insights into athlete behavior, their accuracy may not be consistent across real-world competitive conditions [16,17]. In recent years, emerging technological innovations have sought to overcome the limitations of traditional GPS-based systems. A growing body of evidence suggests that IMU-based or hybrid GPS–IMU devices offer improved sensitivity in detecting short accelerations, decelerations, and micro-movements characteristic of soccer and other field-based sports [12,18]. Multi-sensor platforms, integrating GPS, IMU, gyroscopes, magnetometers, physiological trackers, and impact sensors, demonstrate potential for enhanced measurement fidelity; however, validation evidence remains inconsistent and often limited by small sample sizes, heterogeneous protocols, and insufficient external validation [3].

Practitioner-based research further highlights discrepancies between perceived and actual device accuracy, suggesting that many training-load decisions may rely on data that have not undergone rigorous scientific evaluation. This gap between technological capability and empirical validation has been identified as a significant concern in both research and applied practice, particularly given the increasing reliance on wearable-derived data for training and decision-making [17,19].

Despite the growing body of literature addressing wearable applications in sport, several critical gaps persist. First, validation studies often utilize protocols that fail to capture the complex, intermittent nature of team sports, instead relying on linear running tasks or controlled laboratory tests ill-suited to ecological demands [10,16]. Second, substantial variability exists in gold-standard comparison methods including motion capture systems, radar devices, timing gates, and force platforms complicating cross-study comparisons and synthesis [4,15]. Third, device placement, sensor fusion algorithms, sampling frequency, filtering techniques, and proprietary processing pipelines differ widely among manufacturers, contributing to conflicting findings regarding accuracy and reliability [17,20].

Finally, few studies have examined how wearable accuracy fluctuates across the duration of competitive matches, especially under conditions of fatigue, tactical congestion, or variable environmental stressors that may meaningfully influence real-time measurement validity [8,14]. Given the centrality of wearable-derived metrics in contemporary performance analysis and sports medicine, establishing a rigorous and comprehensive understanding of device validity in team sports represents a critical scientific and practical priority. Misinterpretation of inaccurate or insufficiently validated data may lead to flawed assessments of player load, misguided training interventions, or inadequate injury risk management. Therefore, systematic evaluation of the current evidence base is essential to delineate technological capabilities, identify methodological limitations, and guide the development of improved validation standards.

The present review focuses specifically on wearable-derived physiological monitoring outcomes in team-sport athletes. Heart rate, energy expenditure, VO_2_max, and respiratory frequency were selected because they represent commonly used indicators of internal physiological load, cardiorespiratory response, and metabolic demand in applied sport settings. Although these outcomes differ in their physiological basis, measurement methods, and validation requirements, they are frequently integrated into wearable-based monitoring systems and used by coaches, sports scientists, and medical staff to interpret athlete responses to training and competition. Therefore, this review does not treat wearable-device validity as a single homogeneous construct, but rather examines the strength and limitations of validation evidence separately across these physiological outcome domains.

The aim of this systematic review was to synthesize current evidence on the validity and accuracy of wearable devices used to assess selected physiological monitoring outcomes in team-sport athletes, specifically heart rate, energy expenditure, VO_2_max, and respiratory frequency. Rather than providing a single overall judgment on wearable-device validity, this review aimed to compare the strength, limitations, and methodological characteristics of validation evidence across these outcome domains, with particular attention to reference standards, testing environments, and sport-specific movement demands.

## 2. Materials and Methods

### 2.1. Study Design

This systematic review was conducted in accordance with the Preferred Reporting Items for Systematic Reviews and Meta-Analyses (PRISMA) guidelines [21] to ensure transparency and methodological rigor.

Due to the methodological heterogeneity of the included studies in terms of physiological outcomes, wearable devices, reference standards, and validation protocols, this review was designed as an outcome-specific narrative synthesis. Findings were organized according to predefined physiological outcome domains, heart rate, energy expenditure, and VO_2_max, with respiratory frequency presented as an additional cardiorespiratory measure.

### 2.2. Literature Search Strategy

A systematic literature search was conducted in the PubMed and Scopus databases. Additional studies were identified through manual searches of reference lists and other relevant sources. The search strategy included combinations of keywords and Boolean operators related to wearable technology, validity, and team sports.

In PubMed, the search was performed using combinations of the following keywords: wearable technology, wearable devices, wearable sensors, smartwatch, accelerometer, inertial measurement unit (IMU), GPS, validity, accuracy, reliability, team sports, soccer, basketball, rugby, handball, heart rate, energy expenditure, VO_2_max, and respiratory rate.

An example of the search strategy applied in PubMed is presented below:

(“wearable technology” OR “wearable devices” OR “wearable sensors” OR smartwatch OR “fitness tracker” OR accelerometer OR “inertial measurement unit” OR IMU OR GPS)

AND

(“validity” OR “accuracy” OR “reliability” OR “validation”)

AND

(“team sports” OR soccer OR football OR basketball OR rugby OR handball OR volleyball)

AND

(“heart rate” OR “energy expenditure” OR VO2max OR “oxygen consumption” OR “respiratory rate”)

A similar strategy was adapted for Scopus using title, abstract, and keyword fields.

### 2.3. Inclusion and Exclusion Criteria

Studies were eligible for inclusion if they were original research articles published in English or Serbian within the last ten years (2015–2025). Included studies involved healthy athletes participating in team sports and evaluated wearable devices or microtechnology-based devices measuring at least one physiological parameter relevant to this review, including VO_2_max, heart rate, or energy expenditure. Additionally, studies were required to compare wearable device outputs with a gold-standard reference method and report standard validation metrics.

Studies were excluded if they involved non-athletic or clinical populations, used non-wearable technologies, or assessed outcomes unrelated to the selected physiological parameters. Studies assessing only external-load variables, such as distance covered, acceleration, sprint distance, player load, or positional tracking, without validation of at least one selected physiological outcome, were also excluded. Furthermore, studies were excluded if they lacked comparison with a gold standard, did not report validation statistics, or were published as reviews, meta-analyses, or case reports.

### 2.4. Study Selection

A total of 146 records were identified through database searching (PubMed, n = 62; Scopus, n = 84). After removal of 36 duplicate records, 110 records were screened based on title and abstract, of which 40 were excluded. A total of 70 full-text articles were assessed for eligibility. Following the application of inclusion and exclusion criteria, 61 studies were excluded due to the absence of a sport population (n = 20), lack of a gold-standard comparison (n = 24), absence of validity assessment (n = 15), or inclusion of non-healthy populations (n = 2). Ultimately, 9 studies met the eligibility criteria. In addition, two eligible studies were identified through supplementary screening and reviewer-recommended records during the revision process. Both studies met the predefined inclusion criteria and were added to the final synthesis. Therefore, 11 studies were included in the revised systematic review.

### 2.5. Data Extraction

Data extraction was performed using a standardized form developed for this review. The following information was extracted from each included study: study design, sample characteristics, type of wearable device, gold-standard reference method, validation procedures, and reported validation metrics. Key findings related to the accuracy and reliability of wearable devices were also recorded.

### 2.6. Data Synthesis

Given the heterogeneity of the included studies in terms of device type, physiological outcome, reference standard, testing protocol, and statistical reporting, a meta-analysis was not performed. Findings were synthesized narratively and organized according to physiological outcome domains, including heart rate, energy expenditure, and VO_2_max, and respiratory frequency. Within each domain, studies were compared according to the wearable device used, the reference standard, the testing environment, and the reported validation metrics.

### 2.7. Quality Assessment

The methodological quality of the included studies was assessed using the QUADAS-2 tool, which evaluates the risk of bias and applicability concerns across four domains: patient selection, index test, reference standard, and flow and timing. Each study was rated as having low, high, or unclear risk of bias. The assessment was performed by a single reviewer, and the results were summarized descriptively. Detailed QUADAS-2 assessment results are provided in the Supplementary Materials. Following the inclusion of two additional studies during revision, these studies were assessed using the same QUADAS-2 procedure and added to the Supplementary Materials.

## 3. Results

**Figure 1:**
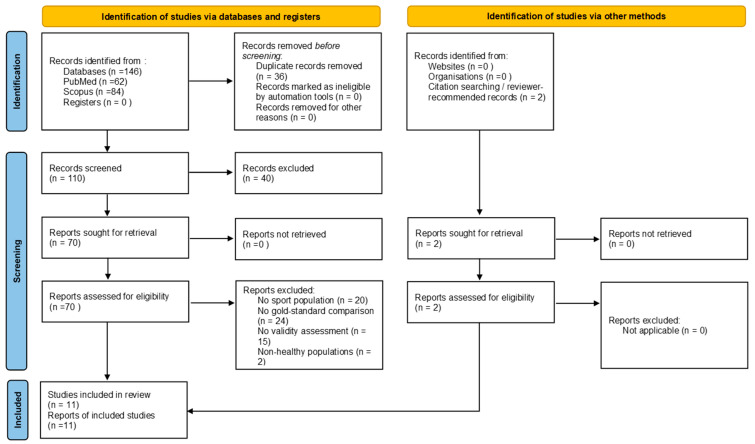
PRISMA flow diagram of the study selection process.

During the study identification process, eleven studies were included after completing the full screening procedure, all presented in Figure 1. All studies met the predefined eligibility criteria and focused on evaluating the validity, reliability, or accuracy of wearable and microtechnology-based devices in team-sport contexts. Devices examined included smartwatches, GPS/IMU systems, local position measurement systems, chest straps, heart-rate monitors, and multi-sensor platforms. The overall methodological quality assessed through the QUADAS-2 tool was predominantly moderate to high across the included studies.

### 3.1. Sample Characteristics

The included studies comprised a total of 188 participants across different team sports and competitive levels. Sample sizes varied across studies, ranging from 8 to 26 participants per study [22,23,24,25,26,27,28,29,30,31,32]. Specifically, Düking et al. [22] included 24 elite youth male footballers, Taylor et al. [23] examined 16 female basketball players, while Montalvo et al. [24] included 22 multi-sport athletes. Similarly, Gastin et al. [25] involved 26 field and court sport athletes, and Di Paco et al. [26] assessed 26 elite football players. Smaller samples were reported in studies such as Martín-Escudero et al. [27] (n = 8) and Costello et al. [28] (n = 10).

The samples predominantly consisted of athletes from soccer, basketball, rugby, and handball with additional representation from multisport populations, including athletics, triathlon, and cross-training [24,27]. All participants were described as trained, competitive, or elite-level athletes.

The age of participants ranged approximately from 17 to 25 years across studies. For example, Düking et al. [22] reported a mean age of 17.3 ± 1.3 years, while Costello et al. [28] included professional rugby players aged 18.1 ± 0.8 years, and Montalvo et al. [24] reported a mean age of 22.1 ± 2.6 years.

Overall, the included samples represent athletic populations assessed under both controlled laboratory conditions and ecologically valid field-based environments.

The characteristics of all included studies are summarized in Table 1.

### 3.2. Validity of Wearable Devices by Physiological Outcome

The validity of wearable and microtechnology-based devices varied depending on the physiological parameter assessed, device type, reference standard, and sport-specific testing context. Therefore, the findings are presented according to the main physiological outcomes examined in the included studies.

#### 3.2.1. Heart Rate

Heart rate monitoring demonstrated the most consistent evidence of acceptable validity across the included studies. Studies involving soccer, basketball, and mixed-sport athletes reported strong agreement between wearable devices and electrocardiography-based or validated reference measurements [24,27]. Device-specific findings indicated that some commercial devices performed better than others. For example, stronger agreement was reported for Apple Watch, Garmin, Polar, and TomTom devices, whereas weaker agreement was observed for Fitbit and Samsung devices, particularly during higher-intensity exercise conditions [24,27].

However, heart-rate validity was not uniform across all testing conditions. Reduced accuracy was reported during high-intensity exercise and complex movement patterns typical of team sports.

#### 3.2.2. Energy Expenditure

Energy expenditure estimation showed consistently low and variable validity across different sports, including rugby, basketball, soccer, handball, and field- or court-sport activities. Most studies reported systematic underestimation of energy expenditure, particularly during intermittent, high-intensity, multidirectional, and collision-based activities [23,25,28,29,30].

Device-specific findings showed that single-sensor approaches were generally insufficient for accurate energy-expenditure estimation. Heart-rate-only and accelerometer-only systems substantially underestimated total energy expenditure in rugby players, while combined sensor models showed comparatively smaller errors but still lacked sufficient individual-level accuracy [28]. Similarly, GPS- or microtechnology-derived metabolic power estimates underestimated energy expenditure during collision-based rugby activity and intermittent running protocols [30,31].

In multidirectional running, metabolic power derived from a 10 Hz microtechnology device underestimated energy expenditure by 52%, compared with 34% during linear running [31]. In team handball, energy expenditure estimated using a local position measurement system was 63–66% lower than spiroergometry-derived values during a game-based performance test, with no significant correlations between methods [32]. Overall, these findings indicate that energy-expenditure estimation remains one of the least accurate physiological outcomes derived from wearable and microtechnology-based systems in team-sport contexts.

#### 3.2.3. VO_2_max

VO_2_max estimation demonstrated mixed validity, primarily assessed in soccer populations, with results depending on the testing protocol and device used. While some agreement with gold-standard measures was observed, variability across trials and testing conditions remained evident [22]. In the included study, smartwatch-derived VO_2_max showed better agreement after the second running trial, whereas the Yo-Yo Intermittent Recovery Test Level 2 showed poor agreement with respiratory gas analysis [22].

#### 3.2.4. Respiratory Frequency

Finally, respiratory frequency measurement, examined in elite soccer players, demonstrated high validity when using a specialized chest-strap device, suggesting that single-parameter wearable systems may provide more accurate measurements compared to multi-sensor devices [26].

Overall, wearable devices appear to demonstrate higher validity in measuring single physiological parameters, while complex estimations such as energy expenditure remain less accurate, particularly in ecologically valid team-sport environments.

## 4. Discussion

The aim of this systematic review was to synthesize current evidence on the validity and accuracy of wearable and microtechnology-based devices used to assess physiological demands in team-sport athletes. The included studies evaluated a range of technologies, including smartwatches, GPS/IMU systems, accelerometers, local position measurement systems, chest-strap sensors, and multi-sensor platforms, with a particular focus on heart rate, energy expenditure, respiratory frequency, and VO_2_max estimation. Overall, the findings reveal substantial variability in measurement accuracy across device categories and testing environments, highlighting the complexity of implementing wearable monitoring tools in high-intensity, intermittent team-sport contexts.

When comparing wearable validity in team sports to findings from endurance-based sports, several similarities and important differences emerge. Endurance disciplines such as distance running and cycling typically demonstrate higher agreement between wearable sensors and gold-standard measures, largely due to the steady-state nature of movement and physiological output. In contrast, team sports involve rapid directional changes, collisions, accelerations, and variable exertion patterns, all of which introduce measurement challenges not present in continuous activities. This is particularly evident in energy-expenditure estimation, where multiple studies in the present review reported substantial underestimation across single-sensor, combined-sensor, GPS-derived, microtechnology-based, and local position measurement approaches [23,25,28,29,30,31,32].

These discrepancies underscore the importance of sport-specific validation, particularly given the movement irregularity and metabolic complexity inherent in team sports. Ecological testing conditions introduce noise, irregular movement patterns, collisions, and rapid intensity fluctuations, all of which can compromise measurement accuracy. For example, field-based movement may lead to GPS signal instability, occlusion during collisions, and inconsistent sampling demands. The findings of Highton et al. [30], who reported approximately 45% underestimation of metabolic power during repeated-effort rugby sessions, illustrate how environment-dependent factors may limit wearable performance in real-world settings. Similar limitations were observed by Oxendale et al. [31], where metabolic power underestimated energy expenditure by 52% during multidirectional running, and by Fuchs et al. [32], where local position measurement underestimated energy expenditure by 63–66% during a team handball game-based performance test.

Laboratory studies, by contrast, generally demonstrate higher validity and narrower limits of agreement [22,23,27,29], indicating that controlled conditions reduce measurement noise. This distinction highlights the importance of differentiating between laboratory-validated performance and real-world field reliability when wearable devices are used in applied settings. Accordingly, validation findings should be interpreted within each outcome domain and testing context rather than generalized across all wearable devices or physiological variables.

Heart-rate monitoring emerged as the most accurate parameter across devices, which aligns with findings from other areas of sports science. The studies included in this review indicate that commercial smartwatches, particularly those equipped with advanced optical sensors, demonstrate strong agreement with electrocardiography-based systems [24,27]. However, reduced accuracy at higher intensities suggests persistent limitations, especially during high-contact or vibration-heavy movements typical of team sports. This reduction in accuracy may be partly explained by the limitations of optical or peripheral pulse-based sensing methods during vigorous movement. Iyriboz et al. [33] reported that pulse oximeter-derived heart-rate readings correlated well with 12-lead ECG at rest and during submaximal exercise, but significantly underestimated heart rate above 155 beats·min^−1^. The authors suggested that probe instability, sweating, exercise-related artefacts, and distortion of the pulse pressure waveform may contribute to reduced accuracy during strenuous exercise. Similar mechanisms may be relevant for wrist-worn optical sensors in team sports, where rapid arm movements, collisions, vibration, and changes in sensor–skin contact can compromise signal quality. In addition, skin pigmentation may influence the accuracy of optical sensing methods, as melanin can affect light absorption and reduce the quality of the reflected photoplethysmographic signal [34]. Therefore, ECG-based systems, particularly 12-lead electrocardiographic recording, remain preferable reference standards for heart-rate validation studies.

A related consideration is the distinction between regulated medical devices and consumer wellness or fitness monitoring tools. Many commercial sport wearables are primarily intended for fitness or wellness feedback rather than clinical cardiac monitoring or medical decision-making. This distinction is relevant because medical devices are subject to specific regulatory and validation requirements, whereas low-risk general wellness products are generally used to support healthy lifestyle or fitness-related feedback [35,36,37]. Therefore, wearable-derived physiological data in sport should be interpreted as supportive monitoring information unless the specific device has been validated and approved for the intended medical use.

In contrast, energy expenditure remains the least accurate physiological metric across wearable categories. Multi-sensor systems consistently underestimate caloric cost, particularly during intermittent high-intensity activity [23,25,28,29,30]. Similar findings were reported in microtechnology- and position-based approaches, with Oxendale et al. [31] showing greater underestimation during multidirectional running than linear running, and Fuchs et al. [32] reporting substantial underestimation during a team handball game-based performance test. These findings reflect broader limitations in predictive modeling, including the inability to fully capture anaerobic contributions, tactical stoppages, changes in direction, collisions, jumps, ball-related actions, and other sport-specific movement patterns.

The development of specialized devices shows promise for improving measurement precision. Di Paco et al. [26] demonstrated excellent agreement between a wearable chest strap and ergospirometry for respiratory frequency. This finding indicates that specialized single-parameter devices may provide high measurement accuracy when the sensor system is specifically designed and validated for the physiological variable being assessed.

Methodological differences across studies further contribute to variability in reported accuracy. Laboratory studies typically use structured protocols that allow for controlled data acquisition, whereas field-based studies introduce variability related to environmental conditions, movement complexity, and athlete behavior. This reflects an ongoing challenge in sports science: balancing ecological validity with measurement precision. For this reason, the findings of the present review should be interpreted as an outcome-specific synthesis rather than as a pooled judgment of wearable-device validity across all physiological variables.

Despite the growing use of wearable technology, several gaps remain in the literature. Few studies have examined device performance across different phases of competition, and long-term monitoring under conditions such as fatigue, dehydration, or environmental stress remains underexplored. Additionally, most validation studies rely on short-duration protocols, limiting insight into cumulative measurement error over time.

These gaps also represent important translational barriers for applied sport settings. A device that demonstrates acceptable validity under controlled laboratory conditions may not maintain the same level of accuracy during training or competition, where movement patterns are less predictable and physiological responses fluctuate rapidly. In addition, proprietary algorithms, differences in sampling frequency, sensor placement, filtering procedures, and manufacturer-specific data-processing pipelines limit comparability across devices and studies. These factors make it difficult for coaches and practitioners to determine whether observed changes reflect true physiological responses or device- and algorithm-related error. Future validation research should therefore prioritize sport-specific protocols, longer monitoring periods, transparent reporting of device algorithms and processing procedures, and multimodal approaches that integrate physiological, movement, and contextual data.

Taken together, the findings of this review indicate that wearable and microtechnology-based devices can provide valuable insights into athlete physiology, particularly for heart-rate and respiratory monitoring. However, the strength of validity evidence differs across physiological outcomes. Heart-rate monitoring showed the most consistent validity, although accuracy may decrease during high-intensity and movement-complex conditions. Energy-expenditure estimation showed the greatest limitations, particularly during intermittent, multidirectional, collision-based, and sport-specific team-sport activities. VO_2_max estimation demonstrated mixed validity and remains dependent on device type and testing protocol.

### Strengths and Limitations

A key strength of this review is the inclusion of studies examining multiple categories of wearable and microtechnology-based devices across both laboratory and field settings, providing a comprehensive overview of current validation evidence. The inclusion of trained and competitive athletes further enhances the practical relevance of the findings.

However, several limitations should be acknowledged. The included studies varied considerably in design, measurement protocols, reference standards, and statistical reporting, limiting direct comparability. Many studies also included relatively small sample sizes, and inconsistent reporting of validation metrics made it difficult to standardize findings across devices. Furthermore, the QUADAS-2 quality assessment was performed by a single reviewer, without independent duplicate assessment or inter-rater reliability verification, which should be considered when interpreting the methodological quality findings. In addition, the number of studies available for each individual physiological outcome was limited, particularly for VO_2_max and respiratory frequency. This heterogeneity limited direct quantitative comparison across studies; therefore, the findings should be interpreted as an outcome-specific narrative synthesis rather than as a pooled estimate of wearable-device validity.

Despite these limitations, this review provides a meaningful foundation for understanding the accuracy of wearable and microtechnology-based monitoring systems in sport and highlights the need for more standardized validation methodologies in future research.

## 5. Conclusions

This systematic review demonstrates that the accuracy of wearable and microtechnology-based devices in team sports depends on device type, physiological outcome, reference standard, and testing environment. Heart-rate monitoring showed the most consistent validity among the examined outcomes, particularly under controlled conditions and when compared with ECG-based or validated reference systems. However, this validity is not uniform across all devices or sport-specific contexts, and accuracy may decrease during high-intensity, intermittent, and movement-complex activities.

Energy-expenditure estimation showed the greatest limitations, with frequent underestimation during multidirectional, collision-based, and sport-specific team-sport conditions. VO_2_max estimation demonstrated mixed validity and remains dependent on device type, testing protocol, and algorithmic assumptions. Respiratory frequency showed high validity in one study using a specialized chest-strap device, but the evidence base remains limited.

Overall, wearable technologies can provide useful information for athlete monitoring, but their outputs should be interpreted as supportive data rather than definitive physiological measures. Future research should prioritize standardized validation protocols, sport-specific testing environments, transparent reporting of device algorithms and processing methods, and stronger comparisons with appropriate reference standards.

## Figures and Tables

**Table 1:** Characteristics of included studies.

Author, Year	Sample	Study Design	Wearable Device/Protocol	Gold Standard	Outcome(s)	Validation Metrics	Key Finding
**Düking et al., 2024 [22]**	N = 24 elite youth male footballers (Tier 3), 17.3 ± 1.3 years	Experimental validation study; comparative protocol (smartwatch vs. YYIR2 vs. gas analysis)	Smartwatch/treadmill ramp test, YYIR2, and two 10-min warm-up runs	Respiratory gas analysis (Cortex Metamax 3B; ramp + verification)	VO_2_max	Run 1: ICC 0.37; MAPE 5.58%; bias −3.16; LOA −15.7 to +9.3. Run 2: ICC 0.54; MAPE 1.06%; bias 0.12; LOA −8.94 to +9.17. YYIR2: ICC 0.17; MAPE 4.2%; bias −2.4; LOA −12.06 to +7.25	Smartwatch validity improves after second run; YYIR2 shows poor agreement with gas analysis
**Taylor et al., 2018 [23]**	N = 16 female NCAA basketball players, 18–23 years	Cross-sectional validation study; 20-m shuttle run + 30-min basketball skills session	SenseWear Mini armband/20-m shuttle run and basketball skills session	Indirect calorimetry (Cosmed K4b2)	EE	Trial I: r = 0.839; SEE = 14.53 kcal; underestimation at higher intensity. Trial II: r = 0.833; SEE = 26.74 kcal; underestimation ≈56.7 kcal	Underestimated EE; error increased with intensity
**Montalvo et al., 2023 [24]**	N = 22 multisport athletes (athletics, football, American football, triathlon, cross-training), 22.1 ± 2.6 years	Experimental validation study; 7 activities (sitting → intervals)	Four commercial smartwatches/seven-activity protocol	HR: Polar H10; EE: COSMED K5	HR, EE	HR: Apple ICC 0.91, r 0.96, MAPE 1.8%, bias −0.77. Garmin ICC 0.83, MAPE 3.5%. Polar ICC 0.81, MAPE 3.9%. Fitbit ICC 0.68, MAPE 6–8%. EE: moderate correlations; high % error (20–35%); wide LOA	Smartwatches accurate for HR (Apple highest); unreliable for EE in athletes
**Costello et al., 2022 [28]**	N = 10 professional rugby players, 18.1 ± 0.8 years	Ecological full-day training validation study	HR-only, ACC-only, and HR+ACC+GPS devices/ecological full-day rugby training	Indirect calorimetry (COSMED K5)	EE	HR-only underestimates TEE by 20–30%; ACC-only 30–50%; combined 10–20% (300–500 kcal/day); wide LOA	All devices substantially underestimate EE; combined sensors best but still unreliable individually
**Gastin et al., 2018 [25]**	N = 26 active field/court sport athletes	90-min session: walk/jog/run + 3 sports circuits	ActiGraph GT3X+; SenseWear SWA/90-min field- and court-sport session	Indirect calorimetry (MetaMax 3B)	EE	GT3X+: bias −29.3%; SWA: −18.2%; circuits −35% to −61%; LOA wide; RMSE ≈140 kJ	Strong EE underestimation during high-intensity intermittent movements
**Di Paco et al., 2024 [26]**	N = 26 elite footballers (Serie A), 23.6 ± 4.8 years	Cross-sectional validation during maximal CPET	Wearable chest strap with strain gauge and HR electrodes/maximal CPET	Breath-by-breath ergospirometry (Vyntus CPX)	fR	r = 0.970; aR^2^ = 0.942; CCC = 0.970; bias 0.17; LOA −4.58 to +4.92; MAE 1.85; RMSE 2.42; ICC 0.97	Very high validity; minimal bias; strong agreement with reference standard
**Martín-Escudero et al., 2023 [27]**	N = 8 competitive athletes (athletics, triathlon, cycling, football)	Laboratory validation during maximal test	Apple Watch; TomTom Runner; Fitbit Charge; Samsung G2/maximal exercise test; HR sampling every 10 s	12-lead ECG	HR	ICC; ARMS; Bland–Altman; APE%; Spearman R; HR underestimated at high intensity	Apple & TomTom most accurate; Fitbit & Samsung weakest at intensities >150 bpm
**Dasa et al., 2022 [29]**	N = 17 professional female footballers, 23.4 ± 3.6 years	Laboratory treadmill validation	Fitbit Charge 3; Polar Vantage V; Garmin 735XT; Apple Watch S4/laboratory treadmill validation	Indirect calorimetry (Vyntus metabolic cart)	EE	R^2^ = 0.956–0.647; SEE = 0.57–1.52 kcal/min; bias = −0.06 to +1.25; LOA wide; RMSE 0.7–2.3	Polar most accurate; Fitbit least accurate; EE varies strongly between devices
**Highton et al., 2017 [30]**	N = 16 rugby players, 23.8 ± 4.8 years	Repeated-effort rugby protocol (sprints + collisions)	Catapult Optimeye S5, 10 Hz GPS + IMU/repeated-effort rugby protocol	Open-circuit spirometry (VO_2_ → EE)	EE	r = 0.63; bias = −5.94 ± 0.67 kcal/min; LOA −6.61 to −5.27	GPS metabolic power underestimates EE by ~45%; poor agreement with calorimetry
**Oxendale et al., 2017 [31]**	N = 12 university-standard team-sport players; rugby, soccer, hockey, and netball; 20.8 ± 2.7 years	Repeated measures validation study; linear and multidirectional intermittent running	MinimaxX 10 Hz microtechnology GPS device/linear and multidirectional running protocol	Indirect calorimetry; Cosmed K4b2 portable gas analyser	EE	r > 0.89, *p* < 0.001; metabolic power underestimated EE by 52% during multidirectional running and 34% during linear running; 95% LoA: 20–93% and 12–59%	Metabolic power substantially underestimated EE, especially during multidirectional running.
**Fuchs et al., 2022 [32]**	N = 11 experienced team handball players; 6 male, 5 female; 25 ± 8 years	Comparative validation study; validated team handball game-based performance test	Catapult ClearSky T6 LPM transponder/team handball game-based performance test	Indirect calorimetry; Cosmed K5 portable spiroergometry system	EE	EELPM was 63–66% lower than EESpiro; no significant correlation for the overall test (r = 0.32, *p* = 0.34) or single heats (r ≤ 0.44)	LPM/metabolic power substantially underestimated EE in sport-specific handball conditions.

Note: HR = heart rate; EE = energy expenditure; fR = respiratory frequency; VO_2_max = maximal oxygen uptake; ICC = intraclass correlation coefficient; LOA = limits of agreement; RMSE = root mean square error; SEE = standard error of estimate; MAPE = mean absolute percentage error; APE = absolute percentage error; ARMS = average root mean square; CCC = concordance correlation coefficient; MAE = mean absolute error; aR^2^ = adjusted coefficient of determination; ACC = accelerometer; GPS = global positioning system; IMU = inertial measurement unit; LPM = local position measurement; CPET = cardiopulmonary exercise testing; YYIR2 = Yo-Yo Intermittent Recovery Test Level 2.

## Data Availability

No new data were created.

## Supplementary Materials

The following supporting information can be downloaded at https://www.mdpi.com/article/10.3390/sports14070264/s1, Table S1: QUADAS-2 quality assessment of included studies.

## References

[1] Li R.T., Kling S.R., Salata M.J., Cupp S.A., Sheehan J., Voos J.E. (2016). Wearable Performance Devices in Sports Medicine. Sports Health.

[2] Bădescu D., Zaharie N., Stoian I., Bădescu M., Stanciu C. (2022). A Narrative Review of the Link between Sport and Technology. Sustainability.

[3] James C., Lam W.-K., Guppy F., Muniz-Pardos B., Angeloudis K., Keramitsoglou I., Knopp M., Ruiz D., Racinais S., Pitsiladis Y.P. (2024). The Integration of Multi-Sensor Wearables in Elite Sport. Sports Sci..

[4] Camomilla V., Bergamini E., Fantozzi S., Vannozzi G. (2018). Trends Supporting the In-Field Use of Wearable Inertial Sensors for Sport Performance Evaluation: A Systematic Review. Sensors.

[5] Zadeh A., Taylor D., Bertsos M., Tillman T., Nosoudi N., Bruce S. (2021). Predicting Sports Injuries with Wearable Technology and Data Analysis. Inf. Syst. Front..

[6] Dellaserra C.L., Gao Y., Ransdell L. (2014). Use of Integrated Technology in Team Sports: A Review of Opportunities, Challenges, and Future Directions for Athletes. J. Strength Cond. Res..

[7] Fox J.L., Scanlan A.T., Stanton R. (2017). A Review of Player Monitoring Approaches in Basketball: Current Trends and Future Directions. J. Strength Cond. Res..

[8] Akenhead R., Nassis G.P. (2016). Training Load and Player Monitoring in High-Level Football: Current Practice and Perceptions. Int. J. Sports Physiol. Perform..

[9] Rampinini E., Alberti G., Fiorenza M., Riggio M., Sassi R., Borges T., Coutts A. (2014). Accuracy of GPS Devices for Measuring High-Intensity Running in Field-Based Team Sports. Int. J. Sports Med..

[10] Alexander J.P., Hopkinson T.L., Wundersitz D.W.T., Serpell B.G., Mara J.K., Ball N.B. (2016). Validity of a Wearable Accelerometer Device to Measure Average Acceleration Values During High-Speed Running. J. Strength Cond. Res..

[11] Sousa A.C., Marques D.L., Marinho D.A., Neiva H.P., Marques M.C. (2023). Assessing and Monitoring Physical Performance Using Wearable Technologies in Volleyball Players: A Systematic Review. Appl. Sci..

[12] Delves R.I.M., Aughey R.J., Ball K., Duthie G.M. (2021). The Quantification of Acceleration Events in Elite Team Sport: A Systematic Review. Sports Med. Open.

[13] Dobbs W.C., Fedewa M.V., MacDonald H.V., Holmes C.J., Cicone Z.S., Plews D.J., Esco M.R. (2019). The Accuracy of Acquiring Heart Rate Variability from Portable Devices: A Systematic Review and Meta-Analysis. Sports Med..

[14] Cheng R., H M Bergmann J. (2022). Impact and workload are dominating on-field data monitoring techniques to track health and well-being of team-sports athletes. Physiol. Meas..

[15] Poitras I., Dupuis F., Bielmann M., Campeau-Lecours A., Mercier C., Bouyer L., Roy J.-S. (2019). Validity and Reliability of Wearable Sensors for Joint Angle Estimation: A Systematic Review. Sensors.

[16] Crang Z.L., Duthie G., Cole M.H., Weakley J., Hewitt A., Johnston R.D. (2021). The Validity and Reliability of Wearable Microtechnology for Intermittent Team Sports: A Systematic Review. Sports Med..

[17] Cardinale M., Varley M.C. (2017). Wearable Training-Monitoring Technology: Applications, Challenges, and Opportunities. Int. J. Sports Physiol. Perform..

[18] Pillitteri G., Thomas E., Battaglia G., Navarra G.A., Scardina A., Gammino V., Ricchiari D., Bellafiore M. (2021). Validity and Reliability of an Inertial Sensor Device for Specific Running Patterns in Soccer. Sensors.

[19] Dawson L., McErlain-Naylor S.A., Devereux G., Beato M. (2024). Practitioner Usage, Applications, and Understanding of Wearable GPS and Accelerometer Technology in Team Sports. J. Strength Cond. Res..

[20] Santos-Gago J.M., Ramos-Merino M., Vallarades-Rodriguez S., Álvarez-Sabucedo L.M., Fernández-Iglesias M.J., García-Soidán J.L. (2019). Innovative Use of Wrist-Worn Wearable Devices in the Sports Domain: A Systematic Review. Electronics.

[21] Page M.J., McKenzie J.E., Bossuyt P.M., Boutron I., Hoffmann T.C., Mulrow C.D., Shamseer L., Tetzlaff J.M., Akl E.A., Brennan S.E. (2021). The PRISMA 2020 Statement: An Updated Guideline for Reporting Systematic Reviews. BMJ.

[22] Düking P., Ruf L., Altmann S., Thron M., Kunz P., Sperlich B. (2024). Assessment of Maximum Oxygen Uptake in Elite Youth Soccer Players: A Comparative Analysis of Smartwatch Technology, Yoyo Intermittent Recovery Test 2, and Respiratory Gas Analysis. J. Sports Sci. Med..

[23] Taylor M., Nagle E., Goss F., Rubinstein E., Simonson A. (2018). Evaluating Energy Expenditure Estimated by Wearable Technology during Variable Intensity Activity in Female Collegiate Athletes. Int. J. Exerc. Sci..

[24] Montalvo S., Martinez A., Arias S., Lozano A., Gonzalez M.P., Dietze-Hermosa M.S., Boyea B.L., Dorgo S. (2023). Commercial Smart Watches and Heart Rate Monitors: A Concurrent Validity Analysis. J. Strength Cond. Res..

[25] Gastin P.B., Cayzer C., Dwyer D., Robertson S. (2018). Validity of the ActiGraph GT3X+ and BodyMedia SenseWear Armband to estimate energy expenditure during physical activity and sport. J. Sci. Med. Sport.

[26] Di Paco A., Bonilla D.A., Perrotta R., Canonico R., Cione E., Cannataro R. (2024). Validity of Wearable Chest Strap for Respiratory Frequency. Sports.

[27] Martín-Escudero P., Cabanas A.M., Dotor-Castilla M.L., Galindo-Canales M., Miguel-Tobal F., Fernández-Pérez C., Fuentes-Ferrer M., Giannetti R. (2023). Are Activity Wrist-Worn Devices Accurate for Determining Heart Rate during Intense Exercise?. Bioengineering.

[28] Costello N., Deighton K., Cummins C., Whitehead S., Preston T., Jones B. (2022). Isolated & Combined Wearable Technology Underestimate the Total Energy Expenditure of Professional Young Rugby League Players; A Doubly Labelled Water Validation Study. J. Strength Cond. Res..

[29] Dasa M.S., Friborg O., Kristoffersen M., Pettersen G., Sundgot-Borgen J., Rosenvinge J.H. (2022). Accuracy of Tracking Devices in Soccer Players. Int. J. Environ. Res. Public Health.

[30] Highton J., Mullen T., Norris J., Oxendale C., Twist C. (2017). Energy Expenditure in Collision-Based Activities. Int. J. Sports Physiol. Perform..

[31] Oxendale C.L., Highton J., Twist C. (2017). Energy expenditure, metabolic power and high speed activity during linear and multi-directional running. J. Sci. Med. Sport.

[32] Fuchs P., Luteberget L.S., Fuchs P.X., Wagner H. (2022). Comparative Analysis of the Indirect Calorimetry and the Metabolic Power Method to Calculate Energy Expenditure in Team Handball. Appl. Sci..

[33] Iyriboz Y., Powers S., Morrow J., Ayers D., Landry G. (1991). Accuracy of pulse oximeters in estimating heart rate at rest and during exercise. Br. J. Sports Med..

[34] Cabanas A.M., Fuentes-Guajardo M., Latorre K., León D., Martín-Escudero P. (2022). Skin Pigmentation Influence on Pulse Oximetry Accuracy: A Systematic Review and Bibliometric Analysis. Sensors.

[35] U.S. Food and Drug Administration (1998). Cardiac Monitor Guidance (Including Cardiotachometer and Rate Alarm): Guidance for Industry.

[36] U.S. Food and Drug Administration (2026). General Wellness: Policy for Low Risk Devices: Guidance for Industry and Food and Drug Administration Staff.

[37] European Parliament and Council of the European Union (2017). Regulation (EU) 2017/745 of 5 April 2017 on Medical Devices. Off. J. Eur. Union.

