# Supplementary material for: How Valid Are Wearable Devices in Team Sports? A Systematic Review
Source: Sports (Basel). 2026 Jun 26;14(7):264. doi: 10.3390/sports14070264 (PMC13416585; doi:10.3390/sports14070264)
Supplement: Supplementary file 1 [file sports-14-00264-s001.zip › sports-4322020-supplementary.pdf]

Author, Year	1. Consecutive sample	2. Case–control avoided	3. No inappropriate exclusions	4. Index test blinded	5. Threshold pre-specified	6. Ref standard accurate	7. Ref standard blinded	8. Appropriate interval	9. All received ref. standard	10. Same ref. standard	11. All included in analysis	Comment
Düking et al., 2024	YES	YES	YES	YES	YES	YES	YES	YES	YES	YES	YES	Low risk across all domains; athletic sample, gold standard of high quality, simultaneous measurement minimizes bias; all participants included in the analysis.
Taylor et al., 2018	YES	YES	YES	YES	YES	YES	YES	YES	YES	YES	YES	Valid design; all participants received the same wearable device and the same gold standard; simultaneous measurement minimizes bias; indirect calorimetry is a precise gold standard; low risk across all domains.
Montalvo et al., 2023	YES	YES	YES	YES	YES	YES	YES	YES	YES	YES	YES	Low risk of bias; athletic population; simultaneous measurement of HR/EE using valid gold standards (Polar

												H10, COSMED K5); identical protocol applied to all participants.
Costello et al., 2022	YES	YES	YES	YES	YES	YES	YES	YES	YES	YES	YES	High-quality study; DLW used as the gold standard; professional rugby players; all participants completed all measurements; simultaneous comparison of SWA and microtechnology; low risk across all domains.
Di Paco et al., 2024	YES	YES	YES	NO	YES	YES	NO	YES	YES	YES	YES	The index test and reference standard were not blinded; other domains showed low risk; elite soccer players; high-quality protocol.
Martín-Escudero et al., 2023	YES	YES	YES	NO	NO	YES	NO	YES	YES	YES	YES	The index test was not blinded; thresholds were not predefined; ECG used as a high-quality reference standard; all participants

												included in the analysis.
Dasa et al., 2022	YES	YES	YES	NO	NO	YES	NO	YES	YES	YES	YES	Wearables were not blinded; thresholds were not predefined; indirect calorimetry used as a reliable gold standard; athletic population; all participants included in the analysis.
Highton et al., 2017	NO	YES	YES	NO	YES	YES	NO	YES	YES	YES	YES	Valid study, but with an expected high risk of bias in domains related to blinding. The experimental design clearly measures the physiological parameter (EE) in an athletic population and is therefore retained in the evidence base.

Gastin et al., 2018	YES	YES	YES	NO	YES	YES	NO	YES	YES	YES	YES	The index test and reference standard were not blinded; indirect calorimetry is a reliable gold standard; athletic population; all participants included; high overall quality except for the expected risk related to blinding.
Oxendale et al., 2017	YES	YES	YES	NO	YES	YES	NO	YES	YES	YES	YES	Valid comparative validation study in team-sport players. The sample and protocol are clearly described, case-control design and inappropriate exclusions were avoided, and all participants received the same index test and reference standard. The 10 Hz micro-technology/GPS -derived metabolic

												power estimate was compared with indirect calorimetry during the same linear and multidirectional running conditions. Expected risk is present for blinding of the index and reference standard, which is common in device-validation studies; applicability is slightly reduced because the protocol was not match play but a team-sport-specific intermittent running protocol.

Fuchs et al., 2022	YES	YES	YES	NO	YES	YES	NO	YES	YES	YES	YES	Strong sport-specific comparative validation study. Experienced team handball players completed a validated game-based performance test while wearing both the Catapult ClearSky T6 LPM transponder and the COSMED K5 spiroergometry system. The index test and reference standard were applied simultaneously to all participants, and indirect calorimetry was an appropriate reference standard. Blinding was not reported, so index-test and reference-standard blinding were rated as NO; overall risk

												remains low to moderate with high applicability for team-handball energy-expenditure validation.
